# A nurse versus a chatbot ‒ the effect of an empowerment program on chemotherapy-related side effects and the self-care behaviors of women living with breast Cancer: a randomized controlled trial

**DOI:** 10.1186/s12912-023-01243-7

**Published:** 2023-04-06

**Authors:** Elham Tawfik, Eman Ghallab, Amel Moustafa

**Affiliations:** 1 Community Health Nursing Department, Faculty of Nursing, Damanhour University, Damanhour, Egypt; 2 Community Health Nursing Department, Faculty of Nursing, The British University in Egypt, Cairo, Egypt; 3 Nursing Education Department, Faculty of Nursing, Alexandria University, Alexandria, Egypt; 4 Medical Surgical Nursing Department, Faculty of Nursing, Galala University, Suez, Egypt

**Keywords:** *Breast c*ancer, Chatbots, Chemotherapy side effects, Conversational agents, MSAS nurse-led education, Patient education, Self-care behaviours

## Abstract

**Background:**

The high levels of unmet needs in relation to provision of self-care information reported by women living with breast cancer suggests that pre-chemotherapy education is suboptimal. Chatbots are emerging as a promising platform to provide education to patients helping them self-manage their symptoms at home. However, evidence from empirical studies on the effect of chatbots education on women living with breast cancer self-care behaviors and symptoms management are scarce.

**Methods:**

This three-arm randomized controlled trial was performed in a chemotherapy day care center within an oncology center in Egypt. A total of 150 women living with breast cancer were randomly selected and randomized into three groups: the ChemoFreeBot group (n = 50), the nurse-led education group (n = 50), and the routine care group (n = 50). In the ChemoFreeBot group, women were given a link to interact with ChemoFreeBot and ask questions about their symptoms and self-care interventions by typing questions or keywords at any time. On the same day as their first day of chemotherapy, the nurse-led education group received face to face teaching sessions from the researcher (nurse) about side effects and self-care interventions. The routine care group received general knowledge during their chemotherapy session about self-care interventions. The self-care behaviors effectiveness and the frequency, severity and distress of chemotherapy side effects were measured at baseline and postintervention for the three groups. The ChemoFreeBot’s usability was assessed.

**Results:**

The mixed design repeated measures ANOVA analyses revealed a statistically significant both group effect and interaction effect of group*time, indicating a significant difference between the three groups in terms of the physical symptoms frequency (F = 76.075, p < .001, F = 147, p < .001, respectively), severity (F = 96.440, p < .001, F = 220.462, p < .001), and distress (F = 77.171, p < .001, F = 189.680, p < .001); the psychological symptoms frequency (F = 63.198, p < .001, F = 137.908, p < .001), severity (F = 62.137, p < .001), (F = 136.740, p < .001), and distress (F = 43.003, p < .001, F = 168.057, p < .001), and the effectiveness of self-care behaviors (F = 20.134, p < .001, F = 24.252, p < .001, respectively). The Post hoc analysis with Bonferroni adjustment in showed that women in the ChemoFreeBot group experienced a statistically significant less frequent, less severe and less distressing physical and psychological symptoms and higher effective self-care behaviors than those in the nurse-led education and routine care groups (p > .001).

**Conclusion:**

ChemoFreeBot was a useful and cost-effective tool to improve increase self-care behavior and reduce chemotherapy side effects in women living with breast cancer through the provision of personalized education and the improvement of the accessibility to real-time and high-quality information compared to “one size fits all” approach used by nurses to provide the information. ChemoFreeBot can be an empowering tool to assist nurses to educate women with breast cancer and allow women to take an active role in managing their symptom.

**Trial registration:**

This study was retrospectively registered in the University hospital Medical Information Network (UMIN) Center, Clinical Trials Registry on 26/09/2022; Registration No:R000055389,Trial ID:UMIN000048955.

**Supplementary Information:**

The online version contains supplementary material available at 10.1186/s12912-023-01243-7.

## Background

In 2020, breast cancer in women was the leading cause of global cancer incidence, with around 2.3 million new cases accounting for one in every four cancer occurrences, representing about 11.7% of all cancer cases [1]. It was the leading cause of cancer-related death in women. An estimated 684,996 women died from breast cancer, with low-resource countries hosting a disproportionately high number of these deaths [2]. Likewise, breast cancer is the most frequent cancer among Egyptian women, accounting for 38.8% of all female cancer cases and more than 22,700 new cases in 2020. Breast cancer has a mortality rate of approximately 11%, making it the second leading cause of cancer-related death after liver cancer [3].

Breast cancer inevitably requires intense treatment with combined modalities. Chemotherapy is one method of treating cancer, with clear evidence of the beneficial effects of this treatment in improving both survival and cancer-related symptoms [4]. However, organ toxicity is a serious problem with chemotherapy, and may result in numerous immediate, short- and long-term side-effects for patients [5]. The severity, frequency, duration, and distress of this toxicity should be evaluated, taking into account both objective and subjective factors [6]. Additionally, the adverse side effects of chemotherapy can negatively impact physical and mental well-being, leading to poor adherence, poor quality of life, morbidity, or even death [7].

Most patients undergoing chemotherapy show signs of suffering from chemotherapy related side effects. However, these side effects are influenced by a number of factors, including the type and amount of chemotherapeutic therapy, the patient’s health, and the stage of cancer [8]. Most cancer patients (60 to 90%) expressed moderate to severe fatigue, 41 to 70% reported disrupted sleep, and 38% indicated significant distress.[9–11] Also, according to a study by Aslam et al. (2014), [8] the most common adverse effects of chemotherapy included weakness (95%), fatigue (90%), nausea (77%), hair loss (76%) and vomiting (75%). Each of these side effects was experienced by more than 70% of the patients. Moreover, the incidence of depression varies between 8% and 36% depending on the site of cancer and diagnostic criteria [12].

Because breast cancer treatment is usually administered in an outpatient setting, more women are managing their condition and treatment at home. Those women must engage in self-care behaviors to control their side effects, lessen their physical and psychological symptom distress, improve their functional status, and maintain their quality of life [13]. Nevertheless, women have reported several barriers and burdens to self-care including lack of time and convenience, high cost, lack of results [14]. and a feeling of powerlessness, which can impede their recovery and ability to resume normal life as they lose control over their health and life, [15, 16] Empowering these women through education about self-care behaviors to relieve the side effects of chemotherapy is imperative. Encouraging them to engage in effective self-care management can help them take an active and positive approach to their cancer experience [17]. Shin and Park’s (2017) [18] study confirmed the moderating effect of participation in self-care behaviors for women living with breast cancer regarding the relationship between their empowerment levels and quality of life.

Education on chemotherapy side effects might reduce health-related suffering by encouraging self-care treatment of such side effects [19]. To educate women living with breast cancer effectively, healthcare practitioners must be knowledgeable about state-of-the-art techniques and interventions [20]. Kessels’ (2003) [21] study showed that 40–80% of cancer sufferers immediately forget the medical information provided by healthcare specialists. Kessels (2003) [21] justified this in terms of the usage of difficult medical terminology, patient-related characteristics including low education levels, and the method of presentation [20]. Nonetheless, the high incidence of patient physical, psychological distress and the need for self-care information suggests that current prechemotherapy preparation is suboptimal, while many studies indicate that patients report high levels of unmet needs in relation to the provision of self-care information. However, many studies have only examined the modifications in patient education that have alleviated particular side- effects, such as oral mucositis, or fatigue [22].

Empowerment education is a new and effective health education model, which can enhance people’s belief to change their unhealthy life behavior [23]. This model is “centered on self-health management, which aims to control disease by stimulating patients’ internal motivation and making them pay more attention to their own health [24]. There is adequate evidence on the advantages of empowerment education, but no agreement about the best way to provide chemotherapy education to women living with breast cancer allowing them to recall the most information. A variety of strategies and techniques can be used to provide adequate empowerment education, which can be offered in a group or individually; face-to face or at distance; led by people with special professional training; and depending on the curriculum. Nevertheless, educational programs may demonstrate different results in clinical and cost-effectiveness [16]. However, a recent review examined the effects of several approaches on the knowledge retention of cancer sufferers. Studies have examined the impact of multifaceted nursing interventions, for instance: face-to-face education sessions, a handbook, an audiotape, and telephone follow-up sessions dramatically decreased symptom intensity and increased self-efficacy in colorectal cancer patients [25].

Nurses have a pivotal role in assisting women in managing the side effects of chemotherapy. They can deliver evidence-based teaching tactics with a patient-centered focus, empowering self-care behaviors and coordinating their care while in treatment. Nevertheless, it can be difficult for nurses to provide women and their families with the overwhelming amount of basic chemotherapy information over a short period of time. Therefore, nurses must discuss strategic solutions for the development of learning types through integration of advanced technologies in various forms of education and conduct more studies to compare the effect of nurse-led education and chat- based education on women’s self-care behaviors for managing chemotherapy - related side effects [26].

Technology-based interventions for the management of cancer are becoming more popular. The utilization of mobile technology and internet-based education for patients have received particular attention in recent years [27, 28]. Information dissemination could become more efficient and timely with the help of technologies like “chatbots.“ A chatbot is an automated text-messaging technology providing patients with information in response to their inquiries [28]. Unlike conventional paper-based discharge instructions, the chatbot’s interface enables providers to give a lot of specific information quickly and on-demand [29, 30]. Studies show that there are positive results for support-chatbots for patients with breast cancer, with an overall satisfaction of 93.95%. Chatbots can be used as virtual assistants, helping their users by playing many different roles, for example symptom checkers, medication reminders or personal data gatherers [31, 32].

Physicians and healthcare professionals appear to be comfortable with using chatbots with most of the automatic simple logistical tasks but find it difficult to accept that they are advanced enough to do complex tasks [31, 33]. However, their usability is limited by the algorithms behind them, their ability to share data, their scalability and the sense of security and privacy they can implement and transmit to their users [32]. To the best of our knowledge, there is limited evidence on the added value of using new technological approaches such as chatbots as a method of educating women living with breast cancer with regard to their self-care behaviors and the management of chemotherapy -related side effects. For this purpose, we created a chatbot, named ChemoFreeBot, as a tool for educating women with breast cancer using the The Microsoft Azure portal. We aimed to examine its effects compared to nurse-led education on the effectiveness of self-care behaviors and the frequency, severity and distress of chemotherapy side effects in among these women. Therefore, we hypothesized that:

1. Women who used ChemoFreeBot would have more effective self-care behaviors and less frequent, severe and distressing physical and psychological chemotherapy side effects than those who received the nurse-led education.
2. Women who used Chemofreebot would have more effective self-care behaviors and less frequent, severe and distressing physical and psychological chemotherapy side effects than those who received the routine care.

## Methods

### Study design and setting

This study was a three-arm randomized controlled trial and was conducted from December 2020 to November 2021 at a chemotherapy day care center within oncology center in El Beheira Governorate, Egypt. It is the only center for oncology patients in this governorate. Therefore, it is characterized by a high flow rate for patients with all types of cancer including breast cancer, lung cancer, head and neck cancer, gastro-intestinal cancer, blood cancer, and cervical cancer. Patients with breast cancer after confirmed diagnosis came to the center to receive either adjuvant or neoadjuvant chemotherapy which was divided into 6 cycles; there was a 21 day rest period between each cycle.

### Participants

The inclusion criteria for participants were as follows: aged 20 years or older, able to read & write, newly diagnosed with breast cancer, and scheduled for their first or second chemotherapy session, non-metastatic cancer stage 0-III, owns a smartphone (personal or shared with their family members) and not receiving any concurrent therapy for any chronic diseases. The study considered including both male and female patients, however, only women did meet the above inclusion criteria at the time of the study.

### Sample size calculation

We calculated post-hoc power of the study based on a mixed design repeated measures ANOVA test comparing average total symptoms frequency and severity scores pre and post intervention with comparison between three programs. We concluded 90% and 92% power for average total symptoms frequency and severity change scores from baseline respectively. We calculated based on effect sizes of 0.25 and 0.35 as well as Pearson’s correlation between repeated measures of 0.119 and 0.079 for the two outcomes respectively at .05 significance level. Power calculation was performed using R software [34].

### Randomization and allocation

Potential participants were selected randomly from a list of women living with breast cancer who are prepared to receive the chemotherapy in the center. Women were assessed using the Karnofsky Performance Scale (KPS) to evaluate their functional abilities on an 11-point rating scale ranging from normal functioning (100) to death (0). Those women whose KPS score was higher than 70%, were asked questions regarding demographic and clinical characteristics data as name, age, education, marital status, occupation, residence, income level, discovery of disease, and stage of disease.

**Fig. 1:**
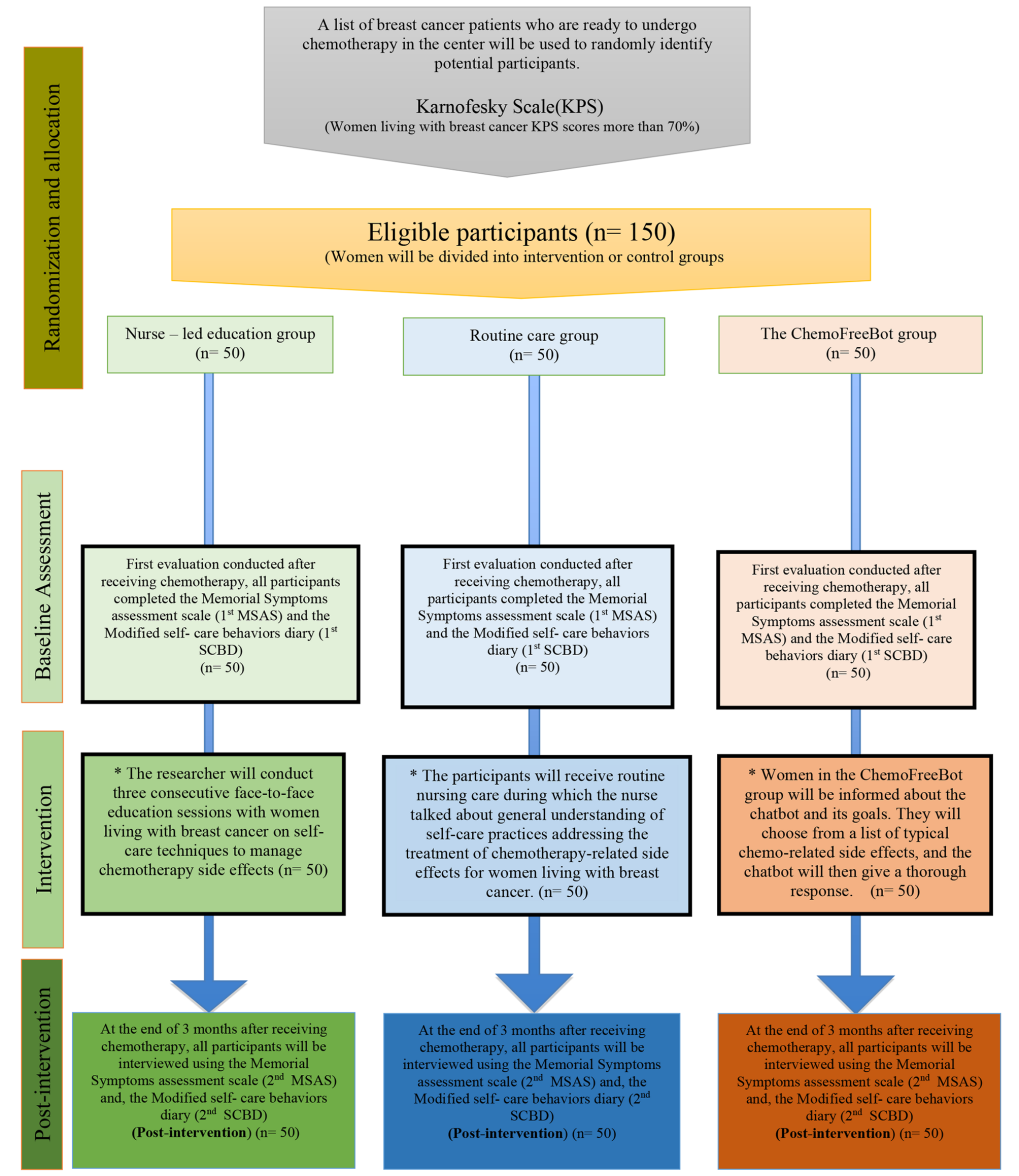
The study flowchart

Cluster by time random sampling technique throughout over months was adopted to allocate the participants either for intervention or control group, one month for each group (Fig. 1). Randomization was performed before baseline assessment. Two independent researchers were responsible for women’s recruitment and random allocation; they were blinded to group allocation. They selected a non-transparent envelope, which contained months’ names and the name of the group either ChemoFreeBot, nurse – led education as intervention groups, or routine care as a control group.

### Intervention

#### The routine care group

The participants in this group received routine nursing care in which the nurse discussed general knowledge of self-care behaviors regarding the management of chemotherapy related side effects to women living with breast cancer. This knowledge varied in depth according to various factors such as nurse workload, teaching ability and the women’s ability to explain their problems and clarify their doubts. Data were collected from this group (over a period of 4 months) from 1/12/2020 until 31/3/2021.

#### The study groups

An empowerment-based intervention in terms of an educational program regarding the management of chemotherapy related side effects to was developed to empower women living with breast cancer. The program was developed to help these women to identify goals and values, understand how their behaviors influenced their health, develop knowledge, skills, and confidence to make decisions about their health enabling them to best achieve their goals, and control chemotherapy related side effects. The intervention was developed and provided for both the nurse-led education and the ChemoFreeBot groups, but in different formats.

##### The nurse-led education group

Three consecutive face to face teaching sessions about self-care behaviors to manage chemotherapy related side effects were delivered to the women by the researcher on the same day as their first day of chemotherapy treatment. The 1st session included information about the definition of cancer, chemotherapy treatment, and the side effects of chemotherapy. The 2nd session included information about self- care behaviors to alleviate such side effects. The 3rd session was a revision of the content delivered in the previous sessions and to answer women’s questions if any. Each session lasted 45 min, separated by a 30-minutes rest break. Each session ranged from 30 to 40 min according to the women’s response. The minimum number of women for each session was 7–10. The researchers used simple clarified media such as PowerPoint to present the educational contents. Each woman received an illustrated color printed brochure developed by the researcher. Data were collected from this group from the period of 1/4/2021 until 31/7/2021.

##### The ChemoFreeBot group

A fully functional and user-friendly chatbot, named ChemoFreeBot, was designed by a team from Microsoft. Microsoft Bot Framework and Azure Bot Service were used to create ChemofreeBot. The chatbot retrieval-based model in a single turn scenario, which only considers the last input message, was used. The researchers developed a Knowledge Base (KB) to respond to queries in natural language. The Knowledge Base was prepared based on the most commonly asked questions, and American cancer society guidelines, 2020. The questions were collected and then divided into categories depending on their type. To add a conversational layer, the developers used the cloud-based API service known as QnA Maker (Cognitive Services QnA Maker). This enabled them to extract Question-Answer (QA) pairs from the data entered into the KB and acquire all the necessary NLP tools for responding to user inquiries. To increase the likelihood of a successful match with a user inquiry, alternative questions were provided. For the users, the chatbot was easy to use; it was a classic chat window where they could type their questions and receive answers in simple language. ChemoFreeBot was designed to be relevant to Android applications because of the widespread popularity and availability of the Android platform on a wide range of devices and operating systems.

Before beginning the RCT, we put ChemoFreeBot through rigorous testing with a pilot of ten women living with breast cancer to assess its effectiveness and enhance its performance. We asked the women to make use of the chatbot in order to evaluate if it could understand their inquiries and address them promptly and correctly, along with reporting any issues that arose so that developers would be able to settle them. This process took five months before it was considered suitable for use in the trial. We wanted to ensure that it operated correctly and accurately before commencing the trial.

Women in the ChemoFreeBot group were taught about the Chatbot and its objectives and explicitly told that they would be chatting to an automated system, not a person. After Chemotherapy, the women received a welcome message through the WhatsApp application and a link to click and begin a dialogue with ChemoFreeBot. They could select from a list of commonly experienced chemotherapy related side effects and the chatbot then provided a detailed answer. Women could interact with ChemoFreeBot at any time. Data were collected from this group from the period of 1/8/2021 until 30/11/2021.

## Outcome measures

### Primary outcomes measures

#### The frequency, severity, and distress of physical and psychological chemotherapy-related side effects

The Memorial Symptoms Assessment Scale (MSAS) was adapted to assess and quantify a large range of physical and psychological symptoms in cancer patients. The MSAS was originally developed by Portenoy et al. (1994) [35] and was utilized to assess the multidimensional experience of symptoms: frequency, severity, and distress of 32 symptoms usually correlated with cancer and its treatment. Patients were asked to demonstrate whether they had encountered each symptom within the previous week. “Frequency” of symptoms was rated as occurring using a 4-point Likert scale (i.e., 1 = rarely, 2 = occasionally, 3 = frequently, and 4 = almost constantly. In addition, “Severity” of symptoms was rated using a 4-point Likert scale (i.e., 1 = slight, 2 = moderate, 3 = severe, 4 = very severe). “Distress” of symptoms was also rated using a 5-point Likert scale (i.e., 0 = not at all, 1 = mild, 2 = moderate, 3 = severe, 4 = very severe). The frequency, severity and distress of chemotherapy side effects were measured at baseline and postintervention for the three groups.

The scoring for the MSAS subsumes several subscale scores: average of frequency, severity, and distress of most the prevalent and perceived physical symptoms (lack of appetite, constipation, diarrhea, mouth sores, nausea, vomiting, change in the way food tastes, changes in skin, dizziness) and was calculated giving the physical symptom subscale score (MSAS- PHYS). The psychological symptom subscale score (MSAS-PSYCH) was calculated from the average of the most prevalent and perceived psychological symptoms within “frequency, severity and distress”: such as worrying, feeling sad, feeling nervous, difficulty sleeping, and feeling irritable. The Total MSAS Score (TMSAS) was the average of the symptom scores of all the most reported symptoms in the MSAS instrument. The score for each symptom was an average of its dimensions. Internal consistency of the PHYS and PSYCH subscale were 0.88 and 0.83, respectively with Cronback alpha coefficients [35].

#### Effectiveness of self-care behaviors

The effectiveness of self- care behaviors for chemotherapy side effects among women living with breast cancer was measured using the Modified Self- Care Behaviors Diary (SCBD). The **SCBD** was originally developed by (Nail et al., 1991) [36]. It is a self-report of the use and effectiveness of self-care behaviors. It contained a checklist of 12 side effects commonly experienced by women receiving parenteral chemotherapy for breast cancer and a list of self-care behaviors aimed at managing these side effects. Content validity of the SCBD was established by Nail et al. and was 0.80 [37]. For the current study, the SCBD was modified by reducing the number of side effects studied to 10. These 10 side effects were the most frequent side effects experienced by women in this study: difficulty sleeping, lack of appetite, constipation, diarrhea, mouth sores, nausea, vomiting, changes in the way food tastes, changes in skin, dizziness, and anxiety and the effectiveness of self-care behaviors for these side effects. Nausea and vomiting self-care measures were grouped together because their self-care behaviors were identical. Also, psychological symptoms (worrying, feeling nervous, feeling sad, and feeling irritable) self-care measures were grouped together for the same reason. The number of self-care behaviors listed ranged from 3 to 17. Because there were variations in the number of self-care behaviors listed for different side effects. The researchers examined the self-care behaviors performed for each side effect as well as the total score for the total number of self-care behaviors. An average score for the number of self-care behaviors used for each side effect was obtained by summing the number of self-care behaviors used and dividing by the number of experienced side effects. The self-care behaviors were measured at baseline and postintervention for the three groups.

### Secondary outcome measure

#### Usability of the chatbot

The Chatbot Usability Questionnaire (CUQ) was used to measure the usability of using chatbots [38]. This scale was composed of 16 validated items aimed to assess the personality, onboarding, navigation, understanding, responses, error handling and intelligence of a chatbot. Women’s levels of agreement with sixteen statements relating to positive and negative aspects of the chatbot, were ranked out of five, from “Strongly Disagree”, to “Strongly Agree”. Final scores were calculated out of 100.

The CUQ is a chatbot-specific usability questionnaire that is equivalent to the Systems Usability Scale (SUS) which is a common instrument used for evaluating systems usability and has a benchmark score of 68 out of a total of 100. The 16 CUQ items were ranked out of five while the scores were calculated out of 80 and then normalized to 100 by dividing the total score of the items by 64 and multiplying the answer by 100. This gave a CUQ score out of 100.

### Data analysis

The quantitative data were described using range (minimum and maximum), mean, standard deviation and median. Calculation of Standard deviation is based on data scattering around the mean and one important measure of dispersion is standard deviation. It is defined as a statistic that measures the dispersion of a dataset relative to its mean and is calculated as the square root of the variance.


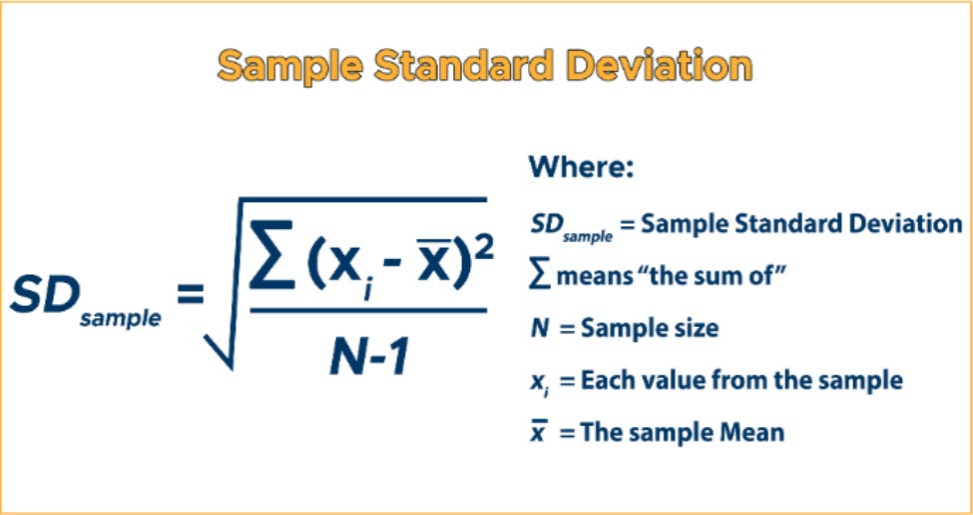


P value was calculated according to each statistical test selected after fulfilling certain assumptions. For example, Chi-Square test was selected to study the association between two categorical variables. One Way ANOVA test was performed to study significant difference in average age between different groups etc. Then p value was set at .05 as a significance level. We added level of significance of .05 at the statistical methods. A level of significance of .05 at the statistical methods was added. Mixed design repeated measures ANOVA test was conducted to study if statistically significant main effect of time, main effect of program whether Chatbot education, Nurse – led education or Routine care and if interaction is present in form of change pattern of different outcome scores along different time between the three groups [39]. All statistical tests were conducted using IBM SPSS statistics program version 28. and R software packages at .05 significance level [34, 40].

## Results

### Baseline socio-demographic and clinical characteristics

The three groups included 150 women with 50 women in each group. Their socio-demographic and clinical characteristics are shown in Table 1. No statistically significant differences were found in the participants’ age, education, marital status, occupation, residence, income level, onset of disease, and the stage of disease (P > .05). The three groups were homogeneous in terms of these variables.

**Table 1:** Women’s baseline socio-demographic and clinical characteristics (n = 150)

Characteristics	ChemoFreeBot (n = 50)		Routine care (n = 50)		Nurse-led education (n = 50)		X^2^/F	p
	No.	%	No.	%	No.	%		
Age								
30 -	14	28.0	13	26.0	14	28.0	4.537 ^a^	0.613 ^c^
40 -	25	50.0	29	58.0	28	56.0		
50 -	7	14.0	6	12.0	8	16.0		
≥ 60	4	8.0	2	4.0	0	0.0		
Min. – Max.	36.0–74.0		36.0–74.0		36.0–55.0		0.897 ^b^	0.410
Mean ± SD.	45.68 ± 8.49		45.38 ± 7.48		43.84 ± 5.90			
**Education**								
Read and write	20	40.0	17	34.0	17	34.0	1.575 ^a^	0.954
Basic education (primary/preparatory)	8	16.0	6	12.0	8	16.0		
Secondary /Diploma	15	30.0	20	40.0	19	38.0		
Higher education	7	14.0	7	14.0	6	12.0		
**Marital status**								
Single	1	2.0	3	6.0	1	2.0	5.947 ^a^	0.410 ^c^
Married	36	72.0	38	76.0	43	86.0		
Divorced	3	6.0	3	6.0	3	6.0		
Widow	10	20.0	6	12.0	3	6.0		
**Occupation**								
Housewife	35	70.0	33	66.0	32	64.0	2.842 ^a^	0.922 ^c^
Technical	0	0.0	1	2.0	0	0.0		
Professional.	11	22.0	10	20.0	13	26.0		
Farmer	4	8.0	6	12.0	5	10.0		
**Residence**								
Urban	26	52.0	32	64.0	22	44.0	4.071 ^a^	0.131
Rural	24	48.0	18	36.0	28	56.0		
**Income sufficiency**								
Not enough	19	38.0	16	32.0	24	48.0	2.738 ^a^	0.254
Enough	31	62.0	34	68.0	26	52.0		
**Onset of cancer (in years)**								
1	21	42.0	13	26.0	17	34.0	4.235 ^a^	0.846 ^c^
2	16	32.0	23	46.0	22	44.0		
3	7	14.0	7	14.0	5	10.0		
4	3	6.0	3	6.0	3	6.0		
5	3	6.0	4	8.0	3	6.0		
**Stage of cancer**								
First	37	74.0	35	70.0	34	68.0	0.450 ^a^	0.798
Second	13	26.0	15	30.0	16	32.0		

Note: SD = standard distribution

^a^ Chi square test

^b^ ANOVA test

^c^ Monte Carlo test

*: Statistically significant at p ≤ .05

### Comparisons of symptom’s frequency, severity, and distress within, among and between groups

The mixed design repeated measures ANOVA analyses revealed a statistically significant time effect, indicating a significant change (a significant difference in time) between the baseline and post-intervention means of the frequency, severity, and distress of both the physical symptoms (F = 147, p < .001, F = 749.679, p < .001, F = 209.281, p < .001, respectively) and psychological symptoms (F = 443.192,p < .001, F = 451.251, p < .001, F = 106.564, p < .001, respectively) across the three groups (Table 2).

**Table 2:** Comparisons of the differences on the frequency, severity, and distress of symptoms within, among and between groups at baseline and post-intervention

Outcomes		ChemoFreeBot (n = 50) mean (SD)	Routine care (n = 50) mean (SD)	Nurse – led education (n = 50) mean (SD)	F(p)	ChemoFreeBot vs. Routine care	ChemoFreeBot vs. Nurse-led education	Routine care vs. Nurse-led education
Symptom frequency								
*Physical* *symptoms*	Baseline	2.87 ± 0.32	2.83 ± 0.29	2.98 ± 0.31	147(p < .001*) ^**a**^			
	Post-intervention	1.36 ± 0.28	2.78 ± 0.23	2.16 ± 0.53	76.075(p < .001*) ^**b**^	**p < .001** ^**^	**p < .001** ^**^	p < .001**
					147(p < .001*) ^**c**^			
*Psychological* *symptoms*	Baseline	2.80 ± 0.38	2.76 ± 0.35	2.76 ± 0.34	443.192(p < .001*) ^**a**^			
	Post-intervention	1.39 ± 0.40	2.00 ± 0.39	2.75 ± 0.30	63.198(p < .001*) ^**b**^	**p < .001** ^**^	**p < .001** ^**^	**p < .001** ^**^
					137.908(p < .001*) ^**c**^			
*Total symptoms*	Baseline	2.84 ± 0.33	2.89 ± 0.25	2.80 ± 0.26	778.765(p < .001*) ^**a**^			
	Post-intervention	1.37 ± 0.30	2.10 ± 0.39	2.77 ± 0.21	97.0(p < .001*) ^**b**^	**p < .001** ^**^	**p < .001** ^**^	**p < .001** ^**^
					230.811(p < .001*) ^**c**^			
Symptom severity								
*Physical* *symptoms*	Baseline	2.87 ± 0.27	2.95 ± 0.23	2.83 ± 0.22	749.679(p < .001*) ^**a**^			
	Post-intervention	1.43 ± 0.29	2.18 ± 0.47	2.80 ± 0.20	96.440(p < .001*) ^**b**^	**p < .001** ^**^	**p < .001** ^**^	**p < .001** ^**^
					220.462(p < .001*) ^**c**^			
*Psychological* *symptoms*	Baseline	2.90 ± 0.32	2.84 ± 0.33	2.81 ± 0.36	451.251(p < .001*) ^**a**^			
	Post-intervention	1.40 ± 0.43	2.06 ± 0.42	2.78 ± 0.33	62.137(p < .001*) ^**b**^	**p < .001** ^**^	**p < .001** ^**^	**p < .001** ^**^
					136.740(p < .001*) ^**c**^			
*Total symptoms*	Baseline	2.88 ± 0.26	2.91 ± 0.22	2.83 ± 0.22	835.079(p < .001*) ^**a**^			
	Post-intervention	1.42 ± 0.30	2.14 ± 0.39	2.79 ± 0.21	111.596(p < .001*) ^**b**^	**p < .001** ^**^	**p < .001** ^**^	**p < .001** ^**^
					247.734(p < .001*) ^**c**^			
Symptom distress								
*Physical* *symptoms*	Baseline	2.89 ± 0.26	2.86 ± 0.29	2.97 ± 0.22	209.281(P < .001*) ^**a**^			
	Post-intervention	1.85 ± 0.40	3.00 ± 0.32	2.76 ± 0.22	77.171(p < .001*) ^**b**^	**p < .001** ^**^	**p < .001** ^**^	**p = 1**
					189.680(p < .001*) ^**c**^			
*Psychological* *symptoms*	Baseline	2.91 ± 0.37	2.96 ± 0.33	2.85 ± 0.34	106.564(p < .001*) ^**a**^			
	Post-intervention	1.68 ± 0.71	2.75 ± 0.33	3.00 ± 0.34	43.003(p < .001*) ^**b**^	**p < .001** ^**^	**p < .001** ^**^	**p = 1**
					168.057(p < .001*) ^**c**^			
*Total symptoms*	Baseline	2.90 ± 0.27	2.93 ± 0.21	2.84 ± 0.26	219.276(p < .001*) ^**a**^			
	Post-intervention	1.80 ± 0.43	2.78 ± 0.20	3.00 ± 0.30	80.265(p < .001*) ^**b**^	**p < .034** ^**^	**p < .001** ^**^	**p = .642**
					234.146(p < .001*) ^**c**^			

F: Mixed design Repeated Measures ANOVA test, SD: Standard deviation, *: Statistically significant at p ≤ .05, ** Significant results after Adjustment for multiple comparisons: Bonferroni

Mixed design repeated measures ANOVA test to assess main effect of time before and after intervention ^a^, main effect of three programs ^b^ and interaction to assess the pattern of change of each quantitative outcome variable along time by program ^c^.

Overall, the frequency and severity of the physical and psychological symptoms decreased in the three groups. With regards to the distress of the physical symptoms, it significantly decreased in the ChemofreeBot and the nurse-led education groups but increased in the routine care group. Likewise, the distress of the psychological symptoms decreased in ChemofreeBot and the routine care groups but increased in the nurse-led education group (Table 2).

Table 2 also shows a statistically significant both group effect and interaction effect of group*time, indicating a significant difference between the three groups in terms of the physical symptoms frequency (F = 76.075, p < .001, F = 147, p < .001, respectively), severity (F = 96.440, p < .001, F = 220.462, p < .001), and distress (F = 77.171, p < .001, F = 189.680, p < .001); and the psychological symptoms frequency (F = 63.198, p < .001, F = 137.908, p < .001), severity (F = 62.137, p < .001, F = 136.740, p < .001), and distress (F = 43.003, p < .001, F = 168.057, p < .001).

The Post hoc analysis with Bonferroni adjustment in Table 2 showed that women in the ChemoFreeBot group experienced a statistically significant less frequent, less severe and less distressing physical and psychological symptoms than those in the nurse-led education and routine care groups (p < .001). Similarly, women in the nurse-led education group reported a statistically significant less frequent and less severe physical and psychological symptoms than those in the routine care group (p < .001). However, both groups did not differ significantly in terms of the physical and psychological symptoms distress level (p = 1).

### Comparisons of self-care behaviors within, among and between groups

The mixed design repeated measures ANOVA analyses revealed a statistically significant time effect, indicating a significant change (a significant difference in time) between the baseline and post-intervention means of the effectiveness of the self-care behaviors that women used to relieve their symptoms (F = 181.752, p < .001) across the three groups. The three groups experienced a statistically significant increase in the effectiveness of the self-care behaviors (Table 3).

**Table 3:** Comparisons of the differences on the effectiveness of self -care behaviors within, among and between groups at baseline and post-intervention

Outcomes		ChemoFreeBot (n = 50) mean (SD)	Routine care (n = 50) mean (SD)	Nurse – led education (n = 50) mean (SD)	F (p)	ChemoFreeBot vs. Routine care	ChemoFreeBot vs. Nurse-led education	Routine care vs. Nurse-led education
Self-care behaviors mean score	Baseline	1.65 ± 0.38	1.85 ± 0.42	1.66 ± 0.43	181.752(p < .001*) ^**a**^			
	Post intervention	2.42 ± 0.49	2.64 ± 0.67	1.81 ± 0.44	20.134(p < .001*) ^**b**^	**p < .001** ^*^	**p < .001** ^*^	**p = .118**
					24.252(p < .001*) ^**c**^			

F: Mixed design Repeated Measures ANOVA test, SD: Standard deviation, *: Statistically significant at p ≤ .05, ** Significant results after Adjustment for multiple comparisons: Bonferroni

Mixed design repeated measures ANOVA test to assess main effect of time before and after intervention ^a^, main effect of three programs ^b^ and interaction to assess the pattern of change of Self-care behaviours mean score variable along time by program ^c^.

Table 3 also shows a statistically significant both group effect and interaction effect of group*time, indicating a significant difference between the three groups with regards to the effectiveness of the self-care behaviors (F = 20.134, p < .001, F = 24.252, p < .001, respectively). The Post hoc analysis with Bonferroni adjustment showed that women in the ChemoFreeBot group reported the highest effectiveness of self-care behaviors among the other groups at postintervention (p < .001) while the nurse-led education and the routine care group did not differ significantly (p = .118).

### Usability of ChemFreeBot

Table 4 reveals that the majority of women in the ChemoFreeBot group reported that the chatbot was easy to use (94% agreed and strongly agreed) and its responses were useful, appropriate and informative (94%). Most also reported that the chatbot understood them well (72%) and was welcoming during the initial setup (88%). Moreover, 70% of the women thought the chatbot was easy to navigate and explained its scope and purpose well. Most of them found that the chatbot’s personality was realistic and engaging (72%) and it coped well with any errors or mistakes they made (76%).

**Table 4:** Usability of ChemoFreeBot

Chatbot usability	Mean ± S D	Chatbot group (n = 50)									
		Strongly Disagree		Disagree		Neutral		Agree		Strongly Agree	
		No.	%	No.	%	No.	%	No.	%	No	%
1. The chatbot’s personality was realistic and engaging	3.64 ± 1.24	6	12.0	0	0.0	14	28.0	18	36.0	18	36.0
2. The chatbot seemed too robotic	1.82 ± 0.66	16	32.0	27	54.0	7	14	0	0.0	0	0.0
3. The chatbot was welcoming during initial setup	4.28 ± 0.67	0	0.0	0	0.0	6	12.0	24	48.0	20	40.0
4. The chatbot seemed very unfriendly	1.96 ± 0.67	12	24.0	28	56.0	10	20.0	0	0.0	0	0.0
5. The chatbot explained its scope and purpose well	4.06 ± 0.82	0	0.0	0	0.0	15	30.0	17	34.0	18	36.0
6. The chatbot gave no indication as to its purpose	1.74± 0.78	23	46.0	17	34.0	10	20.0	0	0.0	0	0.0
7. The chatbot was easy to navigate	3.90 ± 1.31	6	12.0	0	0.0	9	18.0	13	26.0	22	44.0
8. It would be easy to get confused when using the chatbot	1.78 ± 0.65	17	34.0	27	54.0	6	12.0	0	0.0	0	0.0
9. The chatbot understood me well	4.16 ± 0.84	0	0.0	0	0.0	14	28.0	14	28.0	22	44.0
10. The chatbot failed to recognise a lot of my inputs	1.98 ± 0.80	16	32.0	19	38.0	15	30.0	0	0.0	0	0.0
11. Chatbot responses were useful, appropriate and informative	4.20 ± 0.53	0	0.0	0	0.0	3	6.0	34	68.0	13	26.0
12. Chatbot responses were irrelevant	1.90 ± 0.89	22	44.0	11	22.0	17	34.0	0	0.0	0	0.0
13. The chatbot coped well with any errors or mistakes	4.26 ± 0.83	0	0.0	0	0.0	12	24.0	13	26.0	25	50.0
14. The chatbot seemed unable to handle any errors	2.08 ± 0.83	15	30.0	16	32.0	19	38.0	0	0.0	0	0.0
15. The chatbot was very easy to use	4.28 ± 0.57	0	0.0	0	0.0	3	6.0	27	54.0	20	40.0
16. The chatbot was very complex	1.58 ± 0.50	21	42.0	29	58.0	0	0.0	0	0.0	0	0.0
**Total score (Mean ± S D)**	49.94 ± 5.64										
**Total mean score after normalizing (SUS score)**	78.03 ± 8.82										

## Discussion

To the best of our knowledge, there is limited evidence on comparing the effect of chatbots versus nurses as a means of educating women living with breast cancer on the effectiveness of self-care behaviors and chemotherapy side effects. Our multi-arm randomized controlled trial should contribute to closing this gap, with promising results.

The most obvious finding was that ChemoFreeBot had the largest effect on self-care behaviors and the chemotherapy related side effects experienced by women in this study, followed by nurse-led education, whereas routine care had the smallest effect. Women who engaged with the ChemoFreeBot had the most effective self-care behaviors and the lowest physical and psychological symptom frequency, severity and distress at postintervention. This finding suggests that the education provided by ChemofreeBot appears to be more effective in improving these outcomes compared with the education provided by the nurses in the other groups. Although, this finding differs from that of Miles et al. (2021) and Nadarzynski et al. (2021) [41]. that showed that healthcare professionals were perceived as the most suitable and desired source of health-related information, and that chatbots could offer acceptable intervention for less severe conditions and sensitive health issues,it is broadly consistent with others that show the superior effect of chatbots [42]. or its non-inferiority in providing high quality information to patients compared to healthcare professionals [43].

In the current study, the comparison between ChemoFreeBot and nurses is indeed a comparison between two approaches of educating women living with breast cancer about managing chemotherapy side effects. ChemoFreeBot seems to have allowed for a more personalized approach to education that catered for women’s needs, whereas nurses used a “one size fits all” approach whereby women received a uniformly designed general education which may or may not have addressed women’s needs and concerns. Previous literature suggest that cancer patients appear to want the information provided to them to be more thorough and specifically personalized and tailored to their needs [44]. Chatbots are thought to offer information and advice to many people at once, whilst giving the feeling of personalized interaction [45, 46]. The findings of the current study support this idea as ChemoFreeBot appears to have acted as a personal virtual assistant with which the women could personally converse and receive an individually designed information based on their questions. This information was customized and tailored to the women’s individual enquiries with regard to chemotherapy side effects and self-care behaviors. In the usability questionnaire, women reported that ChemoFreeBot understood their enquiries well and the responses they received from it were relevant to what they were asking about. Conversely, women in the other two groups received some general information from the nurse in a single day which was the same day as their chemotherapy treatment, either in the general content-heavy nurse-led education sessions or during the random questions and answers within the routine care in order to manage their symptoms at home.

It has been found that people are more likely to read, process and remember information that is perceived to be personally relevant and tailored to their needs compared to general untailored information [47, 48]. Therefore, it is possible to argue that the individualized education that was provided by ChemoFreebot may have been more effective in allowing the women to focus on and learn better about the symptoms they were experiencing and the self-care intervention that would help to alleviate them. They had an opportunity to read and reread such information at their own pace and as many times as they desired until they fully digested it. Having such personalized learning may have contributed in improving their self-care behaviors and alleviating their symptoms. This is supported by a previous study by Błajda et al. (2022) [49] which also found that personalized education provided through a mobile medical application significantly increased women’s skills and abilities in performing the breast self-examination technique compared to the control group who received general standard education.

Similar to all cancer patients [17, 50, 51], women in the current study experienced chemotherapy side effects at home in the absence of professional support and advice from the nurses or any other healthcare professionals in between their chemotherapy visits. Therefore, in order to be able to manage these side effects when they occurred, it was significant for these women to have the needed information at hand. Research has shown that having access to a reliable source of appropriate and high-quality information at the appropriate time may improve the self-care capacity of women with breast cancer [15, 52]. ChemoFreeBot offered the women unlimited and around the clock access to a high-quality information about chemotherapy side effects and self-care interventions. They were able to directly converse with Chemofreebot when they experienced side effects at home and instantly receive real-time responses to their questions. Women reported that these responses were useful, appropriate and informative. They had instant access, through ChemoFreeBot, to a wide variety of effective, evidence-based self-care interventions from which they could independently select and test until they found which interventions worked for them, hence optimize their self-care behaviors. This may partly explain why women in the ChemoFreeBot group had the highest levels of effective self-care behaviors among the groups. This finding seems to broadly support those of Chaix et al. (2019) [46] who found that the chatbot improved the medication adherence rate of women living with breast cancer by allowing them to access copious information at any time about how to take medications properly, side effects and how to deal with it, as reported by women.

On the other hand, women who were in the nurse led-education and routine care groups had no remote support or follow up phone calls from the nurses or any other healthcare professionals in between their chemotherapy visits. They may have had almost no access to a reliable and valuable source of information when they experienced chemotherapy side effects at home. Consequently, these women may have either relied heavily on their ability to retrieve the general information they received from the nurse on the day of chemotherapy or sought information from other sources such as internet, fellow patients, family, relatives, or friends. Unfortunately, there was no qualitative data in the current study to confirm whether these women used either of these strategies. However, both strategies may be ineffective, and even sometimes counterproductive. For instance, health care professionals expressed concern regarding the validity and reliability of information on the internet or provided by lay people and warned from the negative consequences of misinformation [52–54]. Moreover, relying on the patient’s ability to recall is problematic [55]. Research has shown that patients forget about 50–80% of health information provided to them by health care professionals in healthcare settings as soon as they reach home. Furthermore, about half of what patients remember from this information is incorrect [56]. Thus, it seems possible that these women may have not benefited fully from the information they received from the nurse in day of their chemotherapy treatment.

Having a full access to sufficient, reliable and valuable source information was found to increase women’s living with breast cancer feeling of empowerment and responsibility in managing their symptoms and promoting their own health [15]. Earlier studies show that being better informed about self-care behaviors can reduce chemotherapy-related side effects and the distress caused by them [13, 57, 58]. This also accords with the finding of the current study which showed that women who had instant and full access, through ChemoFreeBot, to evidence-based information about self-care intervention, had the highest effectiveness of self-care behaviors and lowest frequent, severe and distressing physical and psychological symptoms compared to those who did not have such access to information in the other groups. This finding is consistent with the previous studies which reported that having access to evidence-based management strategies of side effects through chatbots caused an improvement in patients’ side effects compared to standard care [22, 59, 60]. For instance, Aranda et al. (2012^) [22]^ reported that those who engaged with the ChemoEd chatbot reported a statistically significant reduction in the prevalence and severity of and bother caused by vomiting, and Greer et al. (2019) [59] reported that participants who used the chatbot experienced a reduction in anxiety after cancer treatment compared to the control group. On other hand, it came as no surprise the findings revealed the distress of the psychological symptoms increased in women who received nurse-led education and the distress of physical symptoms increased in women who received routine care compared to baseline. This finding may suggest that these women may have had difficulty coping with the occurrence of chemotherapy side effects whilst at home. It is possible that these women needed psychological and professional support and desired to feel connected with their nurses and receive reliable information in between chemotherapy visits to help them cope better with their symptoms. A previous study suggested that empowering women living with breast cancer with knowledge and strategies to effectively manage their symptoms would reduce symptoms distress [61].

For many women in this study, visiting the chemotherapy center to seek information or professional help in managing their symptoms from nurses or physicians could be challenging. The commute to the chemotherapy center would take a considerable amount of time and create a physical and financial burden on them, as nearly half of them resides in rural or remote areas far from the chemotherapy center. Based on the findings of the current study, Chemofreebot showed a promise to act as a free-to use information resource that could save these women from visiting healthcare professionals if they had minor side effects that could be managed at home. It could also reduce the need to have a helpline or follow up phone calls that may require large number of staff dedicated to provide consultations to patients. Thus, having a tool such as ChemoFreeBot could contribute to the reduction of financial burden associated with seeking medical care and having in-person consultation, while improving the accessibility to a reliable information that could help alleviating their suffering from chemotherapy side effects.

However, it worth noting that if women’s questions could not be answered by ChemoFreeBot or they experienced complex side effects, women would still need to consult their nurses or physician. Previous studies argued that while chatbots can be an effective tool for providing basic information and answering simple questions of patients, they are still unable to deal with complex problems or understand the complexity of human emotions, and they are unlikely can replace human interaction [41]. This means that ChemoFreeBot cannot replace nurses but rather assist them in supporting and educating women living with breast cancer on the management of side effects after receiving chemotherapy.

### Limitations

This study has a number of limitations that should be noted. First, the study was conducted at a single specialist chemotherapy daycare centre of an oncology centre and only a small number of patients were studied. More research is needed to determine whether this approach can be successfully applied to people with other cancer types and those not treated at specialist centers. Similarly, patients who had previously received chemotherapy were not evaluated in the study. Only patients who had access to the internet and a smart phone could access ChemoFreeBot. Another limitation to be taken into consideration is that there was no mechanism in place to track patient’s use of recommended self-care information at home. Moreover, there was no way to determine how many patients engaged in the suggested self-care activities. Another limitation is that the study lacked some qualitative data about women’ experience of using ChemoFreeBot and the challenges they may have encountered when using it. Future studies should consider using a mixed research design.

## Conclusion

ChemoFreeBot was a useful and cost-effective tool that enabled women living with breast cancer to increase the effectiveness of their self-care behavior and reduce chemotherapy side effects through the provision of personalized education and the improvement of the accessibility to real-time and high-quality information. The “one size fits all” approach used by nurses to provide the information to these was not as effective as the person-centered approach used by ChemoFreeBot. ChemoFreeBot has the potential to be an empowering tool that can be used to assist nurses to educate women with breast cancer and allow women to take an active role in managing their symptom and not remain a passive recipient of information.

## Electronic supplementary material

Below is the link to the electronic supplementary material.

Supplementary Material 1

## Data Availability

All data generated or analysed during this study are included in this published article [and its supplementary information files.
